# What Are the Characteristics of Households That Purchase Alcohol‐Free and Low‐Alcohol Drinks in Great Britain in 2018 and 2021?

**DOI:** 10.1111/dar.70146

**Published:** 2026-04-01

**Authors:** Zoe L. Clarke, Robert Pryce, John Holmes, Abigail K. Stevely, Luke B. Wilson, Inge Kersbergen

**Affiliations:** ^1^ Sheffield Centre for Health and Related Research, University of Sheffield Sheffield UK

**Keywords:** alcohol, alcohol‐free drinks, drinking, low‐strength alcohol, zero‐alcohol drinks

## Abstract

**Introduction:**

The increasing popularity of alcohol‐free and low‐alcohol (NoLo) drinks could reduce alcohol‐related harm if those at greatest risk replace their standard alcoholic drinks with these products. This paper aimed to identify: (i) characteristics associated with occasional purchase of NoLo drinks in Great Britain in 2021; and (ii) whether these characteristics changed between 2018 and 2021.

**Methods:**

Logistic regression using data from Worldpanel by Numerator dataset detailing alcoholic and NoLo drink purchases in the off‐trade (i.e., shops) by households in Great Britain in 2018 (*n* = 14,702) and 2021 (*n* = 15,257). The primary outcome was occasional NoLo purchasing (≥ 4 times in the past year), and secondary outcomes were occasional NoLo beer, cider or wine purchasing.

**Results:**

In 2021, 5.7% of households purchased NoLo ≥ 4 in the past year, compared to 3.0% in 2018. In 2021, these households were more likely to be alcohol purchasers than non‐purchasers [low‐risk purchasers (≤ 112 g per week): OR = 7.11, *p* < 0.001; increasing risk (113–280 g per week): OR = 10.72, *p* < 0.001; higher risk (> 280 g per week): OR = 12.10, *p* < 0.001] and less likely to be from lower social grades than grade AB (Grade C2: OR = 0.72, *p* = 0.008; Grade D: OR = 0.58, *p* < 0.001; Grade E: OR = 0.46, *p* < 0.001). Results were similar for occasional purchasers of NoLo beer, but there was no significant association with social grade for purchasing NoLo cider or wine. The characteristics associated with occasional NoLo purchasing did not change significantly between 2018 and 2021.

**Discussion and Conclusions:**

Households from higher social grades who purchase more alcohol are more likely to regularly purchase NoLo products.

## Key Point Summary

- In 2021, 5.7% of households in the panel dataset in Great Britain purchased no‐ and low‐alcohol drinks (NoLo) four or more times a year.
- These households were more likely to purchase standard alcoholic drinks, with those households buying the most alcohol most likely to purchase NoLo drinks.
- Households occasionally purchasing NoLo drinks were less likely to be from lower social grades.
- Households occasionally purchasing NoLo beer were also more likely to purchase alcohol and less likely to be from lower social grades; however, only purchase of alcohol remained significant for purchasers of NoLo cider and wine.
- The characteristics of occasional NoLo purchasing households did not significantly change between 2018 and 2021.

## 1 Introduction

Over recent years, the market for alcohol‐free and low‐alcohol versions of alcoholic drinks has expanded [1]. The UK Department for Health and Social Care has provided several definitions for these drinks, including ‘alcohol‐free’ for products that do not exceed 0.05% alcohol‐by‐volume (ABV), ‘de‐alcoholised’ for products with an alcohol content of 0.5% ABV and that have had the alcohol removed, and ‘low strength’ for products with an ABV lower than 1.2% [2]. These products are exempt from paying alcohol duty [3]. They are referred to in this paper as NoLo products or drinks.

NoLo drinks accounted for 1.1% of total alcohol sales volume in Great Britain (GB) in 2021, with a value of £221 million [4]. Alcohol‐free products accounted for 82% of NoLo drink sales. NoLo drinks accounted for 0.18% of total alcohol sales value in 2018, which rose to 0.35% in 2021. This is a rapidly growing market, which is expected to continue [5].

Similar to the United Kingdom (UK), the NoLo market is growing in many countries, including the US, Canada and the EU [6, 7]. In the EU, non‐alcoholic beer (≤ 0.5%) has grown from 1.6% sales value of all beer in 2013 to 4.1% in 2019 [7]. The UK's consumption of NoLo beer is lower than in many EU countries, including Germany, Spain, Poland, France and Czechia, which have the highest absolute volume and sold production value of non‐alcoholic beer within the EU.

Increasing the availability of NoLo drinks has been proposed as an intervention for reducing alcohol consumption and improving public health by the UK government [8]. There is potential for reductions in health harm at an individual level if consuming these drinks encourages people to reduce their alcohol intake or to abstain from drinking [1]. However, there is limited evidence to date on whether this is taking place [9]. Concerns have been raised that NoLo normalises alcohol consumption and that the blurring of marketing around NoLo and full‐strength products could lead to consumption of alcohol on new occasions [10].

Health harm experienced from alcohol consumption is unevenly distributed across the population, with those from deprived communities experiencing more harm from alcohol, even when their consumption is at similar levels to those from less deprived communities [11]. For the increased availability of NoLo to be an effective public health intervention and reduce health inequalities, it would need to not only reduce the amount of alcohol consumed by the public, but also for this to occur to a greater degree among those who are most at risk of experiencing harm from alcohol consumption.

Previous studies show that the introduction of new NoLo and mid‐strength beers (containing 1.3% to 3.4% ABV) led to a reduction in grams of alcohol purchased per household in the off‐trade, however this was small in comparison to the reformulation of stronger beers to slightly reduce their strength [12]. Further research found that reductions in grams of alcohol purchased by households that predominantly buy beer in the off‐trade have been offset by increases in grams of alcohol purchased by households that buy wine and spirits [13].

Purchase of NoLo products has been associated with shoppers who are younger, more affluent, from higher socio‐economic groups, and who purchase higher quantities of alcohol [14, 15]. Adoption of NoLo products by people purchasing or drinking more alcohol could be beneficial if they also purchased fewer standard alcoholic drinks. However, the greater acceptance of these products by more affluent groups has the potential to further widen inequalities in alcohol harm.

While previous research has shown characteristics associated with people who have purchased any NoLo products, many of them could be purchasing these products only once, trying a novel product on the market. To be able to explore the potential for NoLo as a health intervention, we need to understand the characteristics of those who regularly buy and drink NoLo products. Previous research has also applied a wider definition of NoLo, which includes mid‐strength products up to 3.5% ABV. This study defines NoLo as products up to 1.2% ABV, as mid‐strength products are more similar to regular strength products and are therefore required to pay alcohol duty [3].

This research addresses this evidence gap by analysing the characteristics of households purchasing NoLo products on an occasional‐to‐regular basis, using a household panel dataset of shoppers in GB. Additionally, this paper will explore whether these characteristics differ by purchased drink types (beer, wine, cider, spirits) and whether there have been any changes in households purchasing NoLo between 2018 and 2021. The study will answer the following research questions:

1. What are the characteristics of households that purchase NoLo drinks four or more times a year in the off‐trade, stratified by product type, compared to those who do not?
2. How have the characteristics of households that purchase NoLo drinks four or more times a year in the off‐trade, stratified by product type, changed from 2018 to 2021?

## 2 Methods

### 2.1 Design

This study uses a repeated cross‐sectional design to compare 2 years of data (2018 and 2021) from a household purchasing panel. The research protocol was pre‐registered on the Open Science Framework [16].

### 2.2 Data

Worldpanel by Numerator is a continuous, household‐level panel dataset collected by the market research company Numerator for commercial purposes and is widely used in UK public health research [17, 18, 19]. The panel comprises N ≈ 30 000 households at any one time. A household member scans the barcode of food and drink products brought into the home. The dataset contains information on the purchasing date, drink type, strength (ABV), volume (ml), price, and whether the product was on offer for each product purchased. It also contains household‐level information, including age group and ethnicity of the main shopper, household size, number of children, social class and household income.

Recruitment to the panel occurs through postal, online and email communication, and panellist population targets are obtained from the results of the BARB Establishment Survey and the Office for National Statistics. Key sample controls (or characteristics of the survey population) include region, household size, presence of children, and age of main shopper. Socio‐economic group is not included in the sample targets but is part of the weightings applied to ensure the survey population is representative of GB. There is continuous replenishment of the sample for attrition purposes. Participants can enter and leave the Worldpanel at any time. Only households that featured in every week of the survey within either 2018 and/or 2021 were included in the sample for analysis, so we could more accurately define occasional purchasers of NoLo products and the purchasing of alcohol. A sensitivity analysis was used to compare the full sample to our selected sample (Table S4).

Worldpanel uses 4‐weekly panel weights to reflect the population of GB. This study used the mean of these weights to create yearly sample weights.

### 2.3 Measures

The primary outcome for this analysis was ‘Occasional NoLo Purchasing’, a binary variable identifying households that purchased NoLo products ≥ 4 times per year. There is no standard definition of occasional purchase for NoLo. This threshold was pragmatically chosen to separate households purchasing NoLo on an occasional to regular basis from households that had *ever* purchased NoLo while still leaving a sufficient number of households to permit detailed analysis. A sensitivity analysis was completed comparing different thresholds (Table S5).

The secondary outcome used was ‘Occasional NoLo Purchasing by Product Type’, containing binary variables determining households who purchased NoLo Beer, NoLo Cider, NoLo Wine, NoLo Spirits and NoLo Ready to Drink (RTD) products ≥ 4 times per year. In 2021, only 15 households occasionally purchased NoLo spirits, and 14 households occasionally purchased NoLo RTD products. Due to small numbers, these product types were not included in the regression analyses.

Independent variables for all analyses were demographic and purchasing characteristics of the household or the main shopper (Table 1). These were: ‘age of main shopper’ (18–28, 28–34, 35–44, 45–54, 55–64, 65+ years), ‘ethnicity of main shopper’ (Asian, Black, Mixed, White, Other, Unknown), ‘region’ (England, Scotland, Wales), ‘social grade’ (categorical variable using National Readership Survey Classifications [20]; AB, C1, C2, D, E—with AB being the most advantaged group and E the least advantaged group) and ‘dependent children’ (children under 18 in the household or not). ‘Weekly alcohol purchase’ was calculated for each adult in the household by totalling units of alcohol purchased for the year, before dividing this by the number of adults living in the household and then dividing by 52 weeks. This was converted to a categorical variable based on NICE thresholds for individual drinking risk [21], including: non‐purchasers (0 grams per week), low‐risk (≤ 112 grams per week), increasing risk (> 112 to ≤ 280 grams per week) and higher risk (> 280 grams per week) purchasers.

**TABLE 1:** Characteristics of sample for 2018 and 2021.

	2018, *n* = 14,702	2021, *n* = 15,257
	%	
Age of main shopper, years		
18–44	**19.0**	**20.7**
45+	**81.1**	**79.4**
Ethnicity of the main shopper		
Asian^a^	**2.1**	**3.1**
Black^b^	**1.3**	**1.2**
Mixed^c^	**0.7**	**0.8**
White^d^	**91.9**	**90.5**
Other	**0.4**	**0.4**
Unknown	**3.6**	**4.1**
Region of household		
England	**86.7**	**86.4**
Scotland	**8.4**	**8.6**
Wales	**4.9**	**5.0**
Social grade		
ABC1	**60.1**	**61.2**
C2DE	**39.9**	**38.8**
Weekly alcohol purchase per adult		
Non‐purchaser	**9.5**	**9.5**
Low risk (≤ 112 g per week)	**79.0**	**76.5**
Increasing risk (> 112 to ≤ 280 g per week)	**8.7**	**10.2**
Higher risk (> 280 g per week)	**2.9**	**3.7**
Dependent children (under 18) in household		
Yes	**24.2**	**25.2**
Number of adults in household		
1–2	**75.1**	**75.7**
3+	**24.9**	**24.3**

*Note:* Bold indicates descriptive statistics.

^a^ Bangladeshi, Chinese, Indian, Pakistani and Asian—other background.

^b^ African, Caribbean and Black—other background.

^c^ Mixed White and Asian, Mixed White and Black African, Mixed White and Black Caribbean, and Mixed—other background.

^d^ White British, White Irish and White—other background. The data for ‘age of main shopper’, ‘social grade’ and ‘number of adults in the household’ were aggregated into a smaller number of categories for use in this table.

Finally, the ‘number of adults in household’ was included in the regressions as a control variable. This is not a variable available in the data, so we calculated this using the household size and number of children variables (Section S6). This was to accurately reflect larger households and to prevent overestimation of the amount of alcohol purchased per week by adults in 3+ households.

### 2.4 Statistical Analysis

1. What are the characteristics of households that purchase NoLo drinks four or more times a year in the off‐trade, stratified by product type, compared to those who do not?

Binary logistic regression was used to explore the characteristics of households that are classified as occasional NoLo purchasers. To correct for familywise errors, the alpha (0.05) was divided by the number of regressions in each research question (*n* = 4), resulting in an alpha of 0.0125 and a 99% confidence interval. Three further binary logistic regressions were then repeated for households occasionally purchasing NoLo Beer, NoLo Cider and NoLo Wine. This was not completed for NoLo Spirits or NoLo RTDs due to the small number of households purchasing these products.

2 How have the characteristics of households that purchase NoLo drinks four or more times a year in the off‐trade, stratified by product type, changed from 2018 to 2021?

A multilevel logistic regression with households clustered within years, due to some households appearing in both 2018 and 2021, and a random intercepts model, was used to analyse whether characteristics of households that occasionally purchased NoLo changed between 2018 and 2021. The alpha was again set to 0.0125 to adjust for familywise error. This analysis was then repeated by product type for households occasionally purchasing NoLo Beer, NoLo Cider and NoLo Wine.

All data were analysed using Stata 18. All analyses were weighted for Research Question 1, but Stata does not allow sample weights in analysis with clustering; therefore, analyses for Research Question 2 were unweighted, and a sensitivity analysis was undertaken by removing the clustering and including weights (Table S2).

## 3 Results

### 3.1 Descriptive Statistics

Only households that met the required eligibility requirements each week for the full duration of the study were included (*n* = 14,702 households in 2018; *n* = 15,257 households in 2021). Sample characteristics for each year are described in Table 1.

In 2021, 5.7% of households purchased NoLo ≥ 4 times within the year. In comparison, only 3.0% of households purchased NoLo ≥ 4 times in 2018. Table 2 describes the percentage of participants who purchased different NoLo products ≥ 4 in that year.

**TABLE 2:** Percentage of sample purchasing of alcohol‐free and low‐alcohol drinks (NoLo) and product types ≥ 4 times per year.

	All NoLo	NoLo Beer	NoLo Cider	NoLo Wine
**2018**	3.0%	1.6%	0.6%	0.7%
**2021**	5.7%	3.3%	1.2%	1.2%

### 3.2 Characteristics of Households Occasionally Purchasing NoLo Drinks (Table 3)

**TABLE 3:** Binary logistic regression exploring characteristics of households occasionally purchasing alcohol‐free and low‐alcohol drinks (NoLo) in 2021.

Predictor	Odds ratio	99% CI	*p* ^a^
Constant	< 0.01	< 0.01, 0.02	< 0.001
Age of the main shopper			
18–28	—	—	—
28–34	1.05	0.22, 4.93	0.942
34–44	0.94	0.21, 4.12	0.910
45–54	0.97	0.22, 4.28	0.951
55–64	0.83	0.18, 3.77	0.750
65+	0.73	0.16, 3.30	0.596
Ethnicity of the main shopper			
Asian^b^	—	—	—
Black^c^	3.06	0.65, 14.51	0.064
Mixed^d^	2.01	0.35, 11.50	0.301
White^e^	2.04	0.75, 5.57	0.067
Other	4.16	0.48, 36.08	0.089
Unknown	1.80	0.52, 6.23	0.225
Region of household			
England	—	—	—
Scotland	1.18	0.84, 1.65	0.215
Wales	1.25	0.75, 2.08	0.253
Social grade			
AB	—	—	—
C1	0.87	0.67, 1.12	0.148
C2	**0.72**	**0.53, 0.99**	**0.008**
D	**0.58**	**0.39, 0.86**	**< 0.001**
E	**0.46**	**0.27, 0.77**	**< 0.001**
Weekly alcohol purchase per adult			
Non‐purchaser	—	—	—
Low risk (≤ 112 g per week)	**7.11**	**2.50, 20.22**	**< 0.001**
Increasing risk (> 112 to ≤ 280 g per week)	**10.72**	**3.68, 31.24**	**< 0.001**
Higher risk (> 280 g per week)	**12.10**	**3.93, 37.24**	**< 0.001**
Dependent children (under 18) in household			
Yes	1.02	0.75, 1.39	0.865
Number of adults in household			
1	—	—	—
2	**1.79**	**1.31, 2.43**	**< 0.001**
3	**1.50**	**1.03, 2.20**	**0.006**
4	**1.81**	**1.03, 3.18**	**0.007**
5	2.08	0.99, 4.36	0.011
6	1.50	0.64, 3.51	0.217

*Note:* Bold indicates a significant result.

Abbreviation: CI, confidence interval.

^a^ Alpha set to 0.01.

^b^ Bangladeshi, Chinese, Indian, Pakistani and Asian—other background.

^c^ African, Caribbean and Black—other background.

^d^ Mixed White and Asian, Mixed White and Black African, Mixed White and Black Caribbean, and Mixed—other background.

^e^ White British, White Irish and White—other background.

Age, ethnicity, region and having dependent children under 18 were not significantly associated with the occasional purchase of NoLo products. Social grade was associated with occasional NoLo purchasing, with those in social grade C2 (odds ratio [OR] = 0.72, *p* = 0.008), D (OR = 0.58, *p* < 0.001), and E (OR = 0.46, *p* < 0.001) significantly less likely to purchase NoLo products than those in social grade AB. Weekly alcohol purchase per adult was significantly associated with occasional purchasing of NoLo, with low risk (OR = 7.11, *p* < 0.001), increasing risk (OR = 10.72, *p* < 0.001) and higher risk (OR = 12.10, *p* < 0.001) purchasers all more likely to purchase NoLo drinks compared to those who purchased no alcohol. Households with two, three, and four adults were significantly more likely to purchase NoLo products than households with only one adult; however, households with five and six adults were not. The model fit was good (Chi^2^ = 168.78; *p* < 0.001).

Occasional NoLo beer purchasers were similar to overall occasional NoLo purchasers: Social grades D (OR = 0.50, *p* = 0.001) and E (OR = 0.50, *p* = 0.007) were significantly less likely to occasionally purchase NoLo Beer than those in higher social grades. Low‐risk (OR = 4.47, *p* = 0.001), increasing risk (OR = 9.03, *p* < 0.001) and higher risk (OR = 10.68, *p* < 0.001) purchasers were significantly more likely to buy NoLo Beer than non‐drinkers. Households with two or three adults were more likely to purchase NoLo Beer than people living alone occasionally.

Social grade was not significant for households who occasionally purchased NoLo cider and NoLo wine; however, the pattern of odds ratios was similar to NoLo beer, suggesting a lack of statistical power. Weekly alcohol purchased was significant, with low risk (0R = 31.47, *p* = 0.001), increasing risk (OR = 28.68, *p* = 0.001), and higher risk (OR = 53.37, *p* < 0.001) purchasers all significantly more likely to occasionally buy NoLo cider than households who did not purchase alcohol. Similarly, occasional purchasing of NoLo wine was associated with being an increasing risk (OR = 10.18, *p* = 0.006) and higher risk (OR = 24.26, *p* < 0.001) purchaser rather than a non‐purchasing household. While ethnicity was not significant in the overall analysis, when looking at those who occasionally purchased NoLo cider, households where the main shopper was from a Black ethnicity were more likely to be purchasers (OR = 35.58, *p* = 0.004) but the confidence interval was extremely wide (99% confidence interval 1.52, 833.34). For both occasional purchases of NoLo cider and NoLo wine, two‐person households were more likely to buy these products than single‐person households. The chi‐square showed good fit in all three models: NoLo beer (Chi^2^ = 161.53, *p* < 0.001); NoLo cider (Chi^2^ = 73.82, *p* < 0.001); and NoLo wine (Chi^2^ = 68.34, *p* < 0.001). Full model results are presented in Table S1.

### 3.3 Changes in NoLo Purchase From 2018 to 2021

The multilevel logistic regression analysis showed a good fit (Chi^2^ = 267.30, *p* < 0.001). However, no significant associations were found, suggesting no substantial changes in the characteristics of occasional NoLo purchasers between 2018 and 2021 (Table 4). In the sensitivity analysis, where clustering was removed and sample weights were applied, one significant association was found (Table S2). This showed that in 2021, households where the main shopper was aged 28–34 years old were more likely to be a purchaser of NoLo than in 2018 (OR = 3.46, *p* = 0.007).

**TABLE 4:** Multilevel logistic regression with years nested by household id and random intercept model for year showing changes in between 2018 and 2021 of households who occasionally purchase alcohol‐free and low‐alcohol drinks.

Predictor	Odds ratio	99% CI	*p* ^a^
Constant	< 0.01	< 0.01, < 0.01	< 0.001
Household‐level variance (SE)	6.68 (0.75)	5.01, 8.91	—
*Age of main shopper* ^a^ *year*			
18–28	1	—	—
28–34	2.75	0.61, 12.40	0.084
34–44	1.07	0.50, 2.29	0.814
45–54	1.41	0.75, 2.65	0.158
55–64	1.27	0.72, 2.23	0.274
65+	1	—	—
*Ethnicity of main shopper* ^a^ *year*			
Asian^b^	—	—	—
Black^c^	4.78	0.20, 116.69	0.207
Mixed^d^	3.96	0.07, 236.30	0.386
White^e^	1.36	0.20, 9.36	0.680
Other	2.74	0.04, 183.86	0.536
Unknown	0.76	0.09, 6.73	0.750
*Region of household* ^a^ *year*			
England	—	—	—
Scotland	1.29	0.63, 2.65	0.353
Wales	1.20	0.46, 3.18	0.625
*Social grade* ^a^ *year*			
AB	—	—	—
C1	1.06	0.64, 1.76	0.773
C2	0.99	0.52, 1.87	0.952
D	0.93	0.44, 1.96	0.800
E	1.24	0.45, 3.41	0.585
*Weekly alcohol purchase per adult* ^a^ *year*			
Non‐purchaser	—	—	—
Low risk (≤ 112 g per week)	2.81	0.66, 11.93	0.066
Increasing risk (> 112 to ≤ 280 g per week)	2.37	0.51, 10.97	0.146
Higher risk (> 280 g per week)	1.85	0.33, 10.20	0.356
*Dependent children (under 18) in household* ^a^ *year*			
Yes	0.10	0.59, 2.05	0.687
Number of adults in household^a^ *year*			
1	—	—	—
2	1.19	0.66, 2.15	0.440
3	1.04	0.48, 2.23	0.905
4	1.19	0.46, 3.10	0.637
5	1.47	0.29, 7.30	0.540
6	1.22	0.21, 6.92	0.772

*Note:* Bold indicates a significant result.

Abbreviation: CI, confidence interval.

^a^ Alpha set to 0.01.

^b^ Bangladeshi, Chinese, Indian, Pakistani and Asian—other background.

^c^ African, Caribbean and Black—other background.

^d^ Mixed White and Asian, Mixed White and Black African, Mixed White and Black Caribbean, and Mixed—other background.

^e^ White British, White Irish and White—other background.

When the analysis was run by product type, models showed good fit: NoLo beer (Chi^2^ = 212.50, *p* < 0.001); NoLo cider (Chi^2^ = 86.38, *p* < 0.001); and NoLo wine (Chi^2^ = 92.40, *p* < 0.001). However, as with the overall model, no significant associations were found (Table S3). In the sensitivity analyses, 2021 households with children were more likely to be occasional purchasers of NoLo cider than in 2018 (OR = 3.20, *p* = 0.008).

### 3.4 Sensitivity Analyses

Our sensitivity analyses showed that compared to the full Worldpanel sample, our selected sample had higher odds of the main shopper being older and having two or three adults in their household, while also having lower odds of being from social grade E (Table S4). Additionally, changing the occasional purchasing threshold to ≥ 3 and ≥ 6 purchases of NoLo per year showed the same pattern of results as our main analysis. See Supporting Information for further details (Table S5).

## 4 Discussion

This study aimed to characterise households that purchase NoLo drinks on an occasional to regular basis and to see if these characteristics have changed over time. We found that 5.7% of households purchased NoLo ≥ 4 per year in 2021, compared to 3.0% in 2018. This appears lower than GB survey data, where 9.8% of participants reported drinking NoLo on a weekly basis in 2022–2023 [22]; however, our study explores data from the off‐trade only.

Our findings show that households in lower social grades are less likely to be occasional purchasers of NoLo products than those in higher grades. This is a graded association, with the odds of being an occasional purchaser reducing as the social grade lowers. These findings are similar to previous studies, which have found that increased purchasing and consumption of NoLo products is a higher socio‐economic phenomenon [14, 15]. This association remained significant for NoLo beer, but not NoLo cider or wine, which is most likely due to a lack of statistical power rather than a different pattern of associations. This is unexpected given the differences in consumption of standard alcoholic drink types by socioeconomic and income group [23, 24].

We also found that occasional household purchase of NoLo is associated with purchasing more alcoholic beverages. Compared to households purchasing no alcohol, those purchasing alcohol were significantly more likely to be occasional NoLo purchasers, with the odds of being an occasional NoLo purchaser increasing with the amount of alcohol purchased. This is in line with previous research in the UK, USA and Finland, where higher levels of alcohol consumption have been found to be significantly associated with NoLo consumption [14, 22, 25].

The literature is mixed on whether age is associated with drinking NoLo products; in Finland, older people were found to be more likely to purchase NoLo beer [14], whereas other UK studies found younger people were more likely to consume NoLo [15, 22]. This analysis did not find any significant associations between age and purchase of NoLo products; it has generally been the case that studies exploring NoLo have found differing age profiles for those consuming these products [22].

Finally, between 2018 and 2021, no significant changes were found in the types of households that were purchasing NoLo drinks in the main analysis. The only changes were found in sensitivity analyses showing that in 2021, main shoppers who were aged 28–34 were more likely to occasionally purchase NoLo, and households with children were more likely to occasionally purchase NoLo Cider than in 2018. This is surprising given the increasing market for NoLo [4, 12], as well as the substantial changes in drinking behaviour during the COVID‐19 pandemic [26]. People from higher socio‐economic backgrounds and those who consume alcohol appear to be most likely to purchase NoLo occasionally.

While it is potentially promising that alcohol consumers, particularly those at increasing and higher risk levels, are adopting NoLo products, it is unclear if this is in addition to or as a replacement for the alcohol they consume. Qualitative research found that some people use NoLo drinks to moderate their alcohol consumption [27, 28]. However, quantitative evidence remains unclear on whether increases in the use of NoLo products could meaningfully contribute to reducing overall levels of alcohol consumption in GB [12, 13].

If the use of NoLo products leads to reduced alcohol consumption, the differences in social grade of those who are purchasing NoLo drinks may widen health inequalities by further improving the health of people in higher social grades. In comparison, the health of those in lower social grades remains unchanged or improves only to a lesser degree. The relatively high price of many NoLo drinks may be a barrier to those in lower socio‐economic groups [29], although there is little evidence available to inform a broader understanding of their attitudes towards these products. Given these results, further research on how NoLo products are perceived by people from lower social grades would be beneficial.

This paper provides valuable new insights into an under‐researched area. This study has benefited from a large, continuously recorded, long‐term data set. This has allowed for the examination of changes in the characteristics of those purchasing NoLo drinks over time. Understanding the characteristics of those who are purchasing NoLo on an occasional to regular basis informs the debate on NoLo as a public health intervention for reducing alcohol use and the potential impact this could have on health inequalities.

There are several limitations to this research. We were interested in looking at the characteristics of those adopting NoLo, rather than those trying these products, to understand the potential of NoLo as a public health intervention. However, there are no standard definitions around the purchase of NoLo, so a pragmatic decision was made on which cut‐off to use. We undertook a sensitivity analysis to support this decision (Table S5), but we acknowledge that purchasing something ≥ 4 times a year may not be considered adoption of a product when compared to other commodities.

Whilst the household panel data used is demographically representative of the GB population and uses weighting methodologies to be reflective of GB purchasing, there are nuances to using panel data that need to be factored in when analysing this dataset. It is, however, comparable to or superior to most other samples used in research on NoLo drinks to date.

An inherent limitation of this research is that we do not know who is drinking alcohol in the household. If the imputed household size is incorrect, or one household member drinks a lot of alcohol while the others do not, the weekly household purchase level will not accurately represent the levels of risky consumption.

Due to the requirement of only including households that met the required eligibility thresholds each week for the full duration of the study, the remaining selected sample was more likely to be older, to have two or three adults in their households, and less likely to be from social grade E. This potentially underestimates true purchasing; however, we control for demographic characteristics in the regression to address this limitation.

Finally, this research focuses on NoLo products bought in the off‐trade and therefore excludes those bought from pubs, bars, or restaurants. However, 81.5% of NoLo by volume is purchased in the off‐trade, so figures apply to the larger market sector [30].

## 5 Conclusions

This paper provides a new understanding of who is buying NoLo products in the off‐trade, with households from higher social grades and who purchase larger amounts of alcohol being more likely to purchase NoLo products on an occasional to regular basis. This has not changed between 2018 and 2021.

## Author Contributions

**Zoe L. Clarke:** formal analysis, investigation, methodology, writing – original draft preparation. **Robert Pryce:** conceptualization, formal analysis, writing – review and editing. **John Holmes:** conceptualization, writing – review and editing, funding acquisition, supervision. **Abigail K. Stevely:** writing – review and editing. **Luke B. Wilson:** writing – review and editing. **Inge Kersbergen:** writing – review and editing, funding acquisition.

## Funding

This project was funded by the National Institute for Health and Care Research (Grant reference number: NIHR135310). Z.L.C. and A.K.S. are funded by the NIHR School for Public Health Research (Grant Reference Number: NIHR204000). The views expressed are those of the authors and not necessarily those of the NIHR or the Department of Health and Social Care.

## Conflicts of Interest

Z.L.C., R.P., A.K.S. and L.B.W. declare no competing interests. J.H. and I.K. have received funding for ongoing, unrelated research on alcohol‐free and low‐alcohol drinks from Alcohol Change UK (ACUK), which received < 0.6% of its funds in 2024–2025 from Lucky Saint, an organisation that produces and sells non‐alcoholic drinks, and owns a pub that sells standard alcoholic drinks. In March 2025, Lucky Saint became an associate member of The Portman Group, a self‐regulatory organisation that is fully funded and controlled by the alcohol industry. ACUK has a strict policy of not accepting any funds from, nor being subject to any influence whatsoever from, the alcohol industry, including through its investment portfolio. ACUK has confirmed that it is in full compliance with this policy.

## Supporting information


**Table S1:** Binary logistic regression exploring characteristics of households occasionally purchasing NoLo by product type in 2021.
**Table S2:** Sensitivity analysis using multilevel logistic regression with sample weights and random intercept model for year showing changes in between 2018 and 2021 of households who occasionally purchase NoLo.
**Table S3:** Multilevel logistic regression with years nested by household id and random intercept model for year showing changes in between 2018 and 2021 of households who occasionally purchase NoLo.
**Table S4:** Sensitivity analysis using binary logistic regression to explore differences in characteristics between all participants in Worldpanel sample compared to chosen sample (households in all 52 weeks of survey).
**Table S5:** Sensitivity analysis using binary logistic regression exploring characteristics of households purchasing NoLo in 2021 to test different cut offs for households regularly purchasing NoLo.

## Data Availability

Use of the Worldpanel by Numerator data is allowed under the terms of the contract and nondisclosure agreement between Numerator and the University of Sheffield, which requires research outputs to be submitted to the data provider ahead of publication. The data providers’ right to request changes is limited to matters of accuracy regarding the data. The data provider played no further role in the research process, including in conception, design, analysis, interpretation, write‐up or the decision to publish.
