# Supplementary material for: What Are the Characteristics of Households That Purchase Alcohol‐Free and Low‐Alcohol Drinks in Great Britain in 2018 and 2021?
Source: Drug Alcohol Rev. 2026 Apr 1;45(4):e70146. doi: 10.1111/dar.70146 (PMC13040626; doi:10.1111/dar.70146)
Supplement: Supplementary file 1 — Table S1: Binary logistic regression exploring characteristics of households occasionally purchasing NoLo by product type in 2021. Table S2: Sensitivity analysis using multilevel logistic regression with sample weights and random intercept model for year showing changes in between 2018 and 2021 of households who occasionally purchase NoLo. Table S3: Multilevel logistic regression with years nested by household id and random intercept model for year showing changes in between 2018 and 2021 of households who occasionally purchase NoLo. Table S4: Sensitivity analysis using binary logistic regression to explore differences in characteristics between all participants in Worldpanel sample compared to chosen sample (households in all 52 weeks of survey). Table S5: Sensitivity analysis using binary logistic regression exploring characteristics of households purchasing NoLo in 2021 to test different cut offs for households regularly purchasing NoLo. [file DAR-45-0-s001.docx]

**Supporting Materials 1**

*Table S1. Binary logistic regression exploring characteristics of households occasionally purchasing NoLo by product type in 2021*

|  | **NoLo Beer Purchasers** | | | **NoLo Cider Purchasers** | | | **NoLo Wine Purchasers** | | |
| --- | --- | --- | --- | --- | --- | --- | --- | --- | --- |
| **Predictor** | **OR** | **99% CI** | **p-value*** | **OR** | **99% CI** | **p-value*** | **OR** | **99% CI** | **p-value*** |
| Constant | <0.01 | <0.01, 0.02 | 0.000 | <0.01 | <0.01, <0.01 | 0.000 | <0.01 | <0.01, 0.43 | 0.000 |
| *Age of main shopper* | | | | | | | | | |
| 18-28 | - | - | - | - | - | - | - | - | - |
| 28-34 | 2.07 | 0.14, 30.64 | 0.486 | 0.46 | 0.08, 2.67 | 0.259 | 0.62 | 0.04, 9.67 | 0.653 |
| 34-44 | 2.37 | 0.17, 32.44 | 0.397 | 0.27 | 0.06, 1.29 | 0.031 | 0.38 | 0.03, 5.08 | 0.340 |
| 45-54 | 1.83 | 0.13, 25.04 | 0.551 | 0.38 | 0.08, 1.85 | 0.113 | 0.64 | 0.05, 8.71 | 0.655 |
| 55-64 | 1.36 | 0.10, 19.46 | 0.764 | 0.32 | 0.06, 1.64 | 0.071 | 0.67 | 0.04, 10.44 | 0.703 |
| 65+ | 1.27 | 0.09, 18.39 | 0.815 | 0.22 | 0.05, 1.09 | 0.015 | 0.66 | 0.04, 10.63 | 0.696 |
| *Ethnicity of main shopper* | | | | | | | | | |
| Asian^1^ | - | - | - | - | - | - | - | - | - |
| Black^2^ | 1.62 | 0.24, 10.86 | 0.514 | **35.58** | **1.52, 833.34** | **0.004** | 0.69 | 0.03, 18.08 | 0.769 |
| Mixed^3^ | 3.59 | 0.39, 33.34 | 0.139 | 6.99 | 0.17, 285.57 | 0.177 | 0.51 | 0.02, 13.72 | 0.596 |
| White^4^ | 2.87 | 0.81, 10.22 | 0.033 | 10.85 | 0.79, 149.29 | 0.019 | 0.83 | 0.12, 5.80 | 0.802 |
| Other | 9.83 | 0.99, 97.57 | 0.010 | 1 | - | - | 1 | - | - |
| Unknown | 2.46 | 0.55, 11.02 | 0.123 | 11.66 | 0.51, 265.69 | 0.043 | 0.35 | 0.03, 4.14 | 0.273 |
| *Region of household* | | | | | | | | | |
| England | - | - | - | - | - | - | - | - | - |
| Scotland | 1.08 | 0.70, 1.67 | 0.665 | 1.08 | 0.52, 2.25 | 0.788 | 1.19 | 0.57, 2.49 | 0.544 |
| Wales | 1.48 | 0.78, 2.81 | 0.119 | 0.59 | 0.19, 1.88 | 0.241 | 1.28 | 0.53, 3.10 | 0.467 |
| *Social grade* | | | | | | | | | |
| AB | - | - | - | - | - | - | - | - | - |
| C1 | 0.79 | 0.57, 1.09 | 0.054 | 1.27 | 0.72, 2.23 | 0.284 | 1.21 | 0.70, 2.10 | 0.372 |
| C2 | 0.67 | 0.46, 1.05 | 0.025 | 0.74 | 0.37, 1.49 | 0.270 | 0.80 | 0.40, 1.60 | 0.403 |
| D | **0.50** | **0.30, 0.85** | **0.001** | 0.87 | 0.37, 2.05 | 0.676 | 0.89 | 0.38, 2.07 | 0.711 |
| E | **0.50** | **0.26, 0.97** | **0.007** | 0.58 | 0.19, 1.83 | 0.222 | 0.46 | 0.14, 1.49 | 0.089 |
| *Weekly alcohol purchase per adult* | | | | | | | | | |
| Non-purchaser | - | - | - | - | - | - | - | - | - |
| Low risk (≤112g per week) | **4.97** | **1.37, 17.98** | **0.001** | **31.47** | **2.33, 424.48** | **0.001** | 8.05 | 0.94, 69.21 | 0.012 |
| Increasing risk (>112 to ≤280g per week) | **9.03** | **2.42, 33.67** | **<0.001** | **28.68** | **2.00, 410.93** | **0.001** | **10.18** | **1.14, 90.82** | **0.006** |
| Higher risk (>280g per week) | **10.68** | **2.67, 42.67** | **<0.001** | **53.37** | **3.40, 836.71** | **<0.001** | **24.26** | **2.65, 221.85** | **<0.001** |
| *Dependent children (under 18) in household* | | | | | | | | | |
| Yes | 0.86 | 0.57, 1.30 | 0.353 | 1.07 | 0.60, 1.92 | 0.760 | 1.18 | 0.63, 2.20 | 0.502 |
| Number of adults in household | | | | | | | | | |
| 1 | - | - | - | - | - | - | - | - | - |
| 2 | **2.04** | **1.34, 3.11** | **<0.001** | **2.32** | **1.16, 4.61** | **0.002** | **2.17** | **1.12, 4.21** | **0.002** |
| 3 | **1.83** | **1.10, 3.04** | **0.002** | 1.78 | 0.78, 4.09 | 0.072 | 1.56 | 0.66, 3.72 | 0.184 |
| 4 | 1.81 | 0.94, 3.50 | 0.020 | 3.26 | 0.98, 10.91 | 0.012 | 1.89 | 0.64, 5.56 | 0.131 |
| 5 | 2.17 | 0.82, 5.74 | 0.040 | 3.42 | 0.90, 12.98 | 0.018 | 2.48 | 0.41, 15.03 | 0.194 |
| 6 | 2.25 | 0.81, 6.27 | 0.041 | 1.24 | 0.13, 11.82 | 0.808 | 2.22 | 0.37, 13.35 | 0.254 |

Bold indicates a significant result. *Alpha set to 0.01; 1. Bangladeshi, Chinese, Indian, Pakistani, and Asian – other background; 2. African, Caribbean, and Black – other background; 3. Mixed White & Asian, Mixed White & Black African, Mixed White & Black Caribbean, and Mixed – other background; 4. White British, White Irish, and White – other background.

Abbreviations: CI, confidence interval; OR, odds ratio.

Source: University of Sheffield Analysis using Kantar’s Worldpanel Take Home Purchase panel 2018 and 2021

**Supporting Materials 2**

*Table S2: Sensitivity analysis using multilevel logistic regression with sample weights and random intercept model for year showing changes in between 2018 and 2021 of households who occasionally purchase NoLo*

| **Predictor** | **OR** | **99% CI** | **p-value*** |
| --- | --- | --- | --- |
| Constant | <0.01 | <0.01, 0.02 | <0.001 |
| *Age of main shopper*year* | | | |
| 18-28 | 1 | - | - |
| 28-34 | **3.46** | **1.06, 11.32** | **0.007** |
| 34-44 | 0.95 | 0.49, 1.84 | 0.845 |
| 45-54 | 1.42 | 0.84, 2.38 | 0.085 |
| 55-64 | 1.07 | 0.66, 1.73 | 0.730 |
| 65+ | 1 | - | - |
| *Ethnicity of main shopper*year* | | | |
| Asian^1^ | - | - | - |
| Black^2^ | 2.99 | 0.19, 46.91 | 0.305 |
| Mixed^3^ | 0.86 | 0.03, 25.13 | 0.908 |
| White^4^ | 0.63 | 0.12, 3.38 | 0.477 |
| Other | 1.47 | 0.04, 56.25 | 0.785 |
| Unknown | 0.53 | 0.08, 3.68 | 0.402 |
| *Region of household *year* | | | |
| England | - | - | - |
| Scotland | 1.23 | 0.68, 2.24 | 0.374 |
| Wales | 1.29 | 0.53, 3.10 | 0.461 |
| *Social grade*year* | | | |
| AB | - | - | - |
| C1 | 1.14 | 0.74, 1.76 | 0.449 |
| C2 | 1.14 | 0.66, 1.96 | 0.546 |
| D | 1.22 | 0.63, 2.34 | 0.435 |
| E | 1.02 | 0.42, 2.47 | 0.961 |
| *Weekly alcohol purchase per adult*year* | | | |
| Non-purchaser | - | - | - |
| Low risk (≤112g per week) | 1.61 | 0.35, 7.34 | 0.418 |
| Increasing risk (>112 to ≤280g per week) | 1.19 | 0.25, 5.74 | 0.774 |
| Higher risk (>280g per week) | 1.06 | 0.20, 5.71 | 0.929 |
| *Dependent children (under 18) in household*year* | | | |
| Yes | 0.93 | 0.54, 1.59 | 0.729 |
| Number of adults in household**year* | | | |
| 1 | - | - | - |
| 2 | 1.13 | 0.68, 1.86 | 0.541 |
| 3 | 0.89 | 0.47, 1.66 | 0.622 |
| 4 | 1.18 | 0.51, 2.69 | 0.616 |
| 5 | 1.07 | 0.32, 3.59 | 0.890 |
| 6 | 1.60 | 0.41, 6.20 | 0.374 |

Bold indicates a significant result. *Alpha set to 0.01; 1. Bangladeshi, Chinese, Indian, Pakistani, and Asian – other background; 2. African, Caribbean, and Black – other background; 3. Mixed White & Asian, Mixed White & Black African, Mixed White & Black Caribbean, and Mixed – other background; 4. White British, White Irish, and White – other background.

Abbreviations: CI, confidence interval; OR, odds ratio.

Source: University of Sheffield Analysis using Kantar’s Worldpanel Take Home Purchase panel 2018 and 2021.

**Supporting Materials 3**

*Table S3: Multilevel logistic regression with years nested by household id and random intercept model for year showing changes in between 2018 and 2021 of households who occasionally purchase NoLo*

|  | **NoLo Beer Purchasers** | | | **NoLo Cider Purchasers** | | | **NoLo Wine Purchasers** | | |
| --- | --- | --- | --- | --- | --- | --- | --- | --- | --- |
| **Predictor** | **OR** | **99% CI** | **p-value*** | **OR** | **99% CI** | **p-value*** | **OR** | **99% CI** | **p-value*** |
| Constant | <0.01 | <0.01, <0.01 | <0.001 | <0.01 | <0.01, 0.03 | <0.001 | <0.01 | <0.01, 0.02 | <0.001 |
| Household-level variance (SE) | 6.41 (0.69) | 4.86, 8.45 | - | 5.20 (0.87) | 3.38, 8.01 | - | 6.06 (0.91) | 4.11, 8.96 | - |
| *Age of main shopper*year* | | | | | | | | | |
| 18-28 | 1 | - | - | 1 | - | - | 1 | - | - |
| 28-34 | 3.84 | 0.51, 29.05 | 0.087 | 1.11 | 0.96, 12.93 | 0.912 | 1 | - | - |
| 34-44 | 1.11 | 0.43, 2.84 | 0.771 | 0.55 | 0.13, 2.23 | 0.267 | 2.54 | 0.52, 12.55 | 0.132 |
| 45-54 | 1.24 | 0.55, 2.79 | 0.487 | 1.03 | 0.34, 3.15 | 0.948 | 2.50 | 0.71, 8.74 | 0.059 |
| 55-64 | 1.01 | 0.49, 2.08 | 0.981 | 1.05 | 0.36, 3.04 | 0.906 | 1.03 | 0.39, 2.71 | 0.930 |
| 65+ | 1 | - | - | 1 | - | - | 1 | - | - |
| *Ethnicity of main shopper*year* | | | | | | | | | |
| Asian^1^ | - | - | - | - | - | - | - | - | - |
| Black^2^ | 0.61 | 0.01, 42.01 | 0.766 | 1 | - | - | 0.95 | <0.01, 235.60 | 0.980 |
| Mixed^3^ | 1 | - | - | 1.51 | <0.01, 501.24 | 0.854 | 1 | - | - |
| White^4^ | 0.64 | 0.03, 15.54 | 0.720 | 4.38 | 0.08, 239.78 | 0.342 | 1.82 | 0.05, 68.03 | 0.671 |
| Other | 1 | - | - | 1 | - | - | 1 | - | - |
| Unknown | 0.41 | 0.01, 12.52 | 0.501 | 2.86 | 0.03, 245.99 | 0.543 | 0.52 | 0.01, 35.26 | 0.693 |
| *Region of household* *year | | | | | | | | | |
| England | - | - | - | - | - | - | - | - | - |
| Scotland | 1.24 | 0.49, 3.13 | 0.549 | 0.81 | 0.22, 2.95 | 0.668 | 1.68 | 0.39, 7.27 | 0.362 |
| Wales | 1.28 | 0.37, 4.46 | 0.615 | 0.37 | 0.07, 2.03 | 0.132 | 2.92 | 0.41, 20.90 | 0.161 |
| *Social grade**year | | | | | | | | | |
| AB | - | - | - | - | - | - | - | - | - |
| C1 | 0.83 | 0.44, 1.58 | 0.464 | 1.48 | 0.56, 3.95 | 0.300 | 2.00 | 0.77, 5.18 | 0.061 |
| C2 | 0.76 | 0.34, 1.72 | 0.387 | 0.97 | 0.29, 3.23 | 0.950 | 1.30 | 0.38, 4.40 | 0.584 |
| D | 1.17 | 0.40, 3.39 | 0.704 | 0.98 | 0.26, 3.62 | 0.967 | 1.24 | 0.33, 4.68 | 0.679 |
| E | 1.43 | 0.38, 5.40 | 0.492 | 0.94 | 0.15, 5.86 | 0.925 | 1.58 | 0.25, 10.01 | 0.525 |
| *Weekly alcohol purchase per adult**year | | | | | | | | | |
| Non-purchaser | - | - | - | - | - | - | - | - | - |
| Low risk (≤112g per week) | 3.30 | 0.63, 17.13 | 0.062 | 8.31 | 0.35, 195.07 | 0.084 | 0.88 | 0.03, 29.22 | 0.922 |
| Increasing risk (>112 to ≤280g per week) | 5.26 | 0.88, 31.33 | 0.017 | 5.53 | 0.20, 152.22 | 0.184 | 0.74 | 0.02, 27.41 | 0.830 |
| Higher risk (>280g per week) | 4.05 | 0.52, 31.37 | 0.078 | 6.39 | 0.17, 242.33 | 0.189 | 1.05 | 0.02, 45.02 | 0.972 |
| *Dependent children (under 18) in household*year* | | | | | | | | | |
| Yes | 1.10 | 0.50, 2.39 | 0.758 | 2.91 | 0.89, 9.50 | 0.020 | 0.49 | 0.14, 1.71 | 0.140 |
| Number of adults in household*year | | | | | | | | | |
| 1 | - | - | - | - | - | - | - | - | - |
| 2 | 1.37 | 0.64, 2.93 | 0.280 | 0.93 | 0.28, 3.09 | 0.881 | 2.01 | 0.69, 5.86 | 0.091 |
| 3 | 1.80 | 0.66, 4.89 | 0.131 | 0.71 | 0.17, 2.96 | 0.531 | 1.27 | 0.32, 5.00 | 0.654 |
| 4 | 2.79 | 0.73, 10.69 | 0.049 | 0.52 | 0.10, 2.79 | 0.321 | 1.89 | 0.29, 12.44 | 0.387 |
| 5 | 3.01 | 0.42, 21.76 | 0.151 | 0.52 | 0.03, 10.39 | 0.575 | 4.42 | 0.11, 174.68 | 0.298 |
| 6 | 2.15 | 0.20, 22.92 | 0.405 | 1 | - | - | 1.34 | 0.07, 26.65 | 0.801 |

Bold indicates a significant result. *Alpha set to 0.01; 1. Bangladeshi, Chinese, Indian, Pakistani, and Asian – other background; 2. African, Caribbean, and Black – other background; 3. Mixed White & Asian, Mixed White & Black African, Mixed White & Black Caribbean, and Mixed – other background; 4. White British, White Irish, and White – other background.

Abbreviations: CI, confidence interval; OR, odds ratio.

Source: University of Sheffield Analysis using Kantar’s Worldpanel Take Home Purchase panel 2018 and 2021.

**Supporting Materials 4**

To explore the differences between the full sample for 2018 and 2021 with the selected sample (households in all 52 weeks of the survey) a logistic regression was performed, comparing samples by the independent variables used in this study. Weekly alcohol purchase per adult was omitted from this regression due to it being calculated based on the assumption that households were present in all 52 weeks. In both 2018 and 2021, the selected sample had statistically higher odds of the main shopper being older and having two or three adults in their household, while also having lower odds of being from social grade E, compared to the full sample. In 2018, the selected sample were also significantly more likely to have four adults living in the household and for the main shopper to be of Black or White ethnicity.

*Table S4: Sensitivity analysis using binary logistic regression to explore differences in characteristics between all participants in KWP sample compared to chosen sample (households in all 52 weeks of survey)*

|  | **Testing 2018 sample** | | | **Testing 2021** | | |
| --- | --- | --- | --- | --- | --- | --- |
| **Predictor** | **OR** | **99% CI** | **p-value*** | **OR** | **99% CI** | **p-value*** |
| Constant | 0.01 | <0.01, 0.18 | <0.001 | 0.03 | 0.02, 0.04 | <0.001 |
| *Age of main shopper* | | | | | | |
| 18-28 | - | - | - | - | - | - |
| 28-34 | **5.19** | **3.15, 8.55** | **<0.001** | **3.27** | **2.30, 4.63** | **<0.001** |
| 34-44 | **14.44** | **8.87, 23.49** | **<0.001** | **7.15** | **5.11, 10.01** | **<0.001** |
| 45-54 | **22.93** | **14.11, 37.27** | **<0.001** | **11.21** | **8.01, 15.66** | **<0.001** |
| 55-64 | **28.00** | **17.21, 45.55** | **<0.001** | **13.80** | **9.86, 19.32** | **<0.001** |
| 65+ | **30.92** | **19.01, 50.28** | **<0.001** | **13.85** | **9.90, 19.38** | **<0.001** |
| *Ethnicity of main shopper* | | | | | | |
| Asian^1^ | - | - | - | - | - | - |
| Black^2^ | **1.47** | **1.12, 1.93** | **<0.001** | 0.88 | 0.68, 1.15 | 0.216 |
| Mixed^3^ | 1.37 | 0.98, 1.90 | 0.016 | 0.98 | 0.72, 1.33 | 0.853 |
| White^4^ | **1.67** | **1.42, 1.97** | **<0.001** | 1.13 | 0.96, 1.31 | 0.021 |
| Other | 1.30 | 0.85, 1.99 | 0.111 | 0.92 | 0.61, 1.38 | 0.589 |
| Unknown | 1.08 | 0.88, 1.33 | 0.335 | 0.85 | 0.71, 1.02 | 0.020 |
| *Region of household* | | | | | | |
| England | - | - | - | - | - | - |
| Scotland | 0.93 | 0.85, 1.02 | 0.057 | 0.96 | 0.88, 1.05 | 0.249 |
| Wales | 0.92 | 0.82, 1.04 | 0.076 | 0.95 | 0.84, 1.06 | 0.223 |
| *Social grade* | | | | | | |
| AB | - | - | - | - | - | - |
| C1 | 1.03 | 0.96, 1.11 | 0.224 | 1.00 | 0.93, 1.07 | 0.970 |
| C2 | 0.99 | 0.91, 1.07 | 0.702 | 0.97 | 0.89, 1.05 | 0.275 |
| D | 1.00 | 0.91, 1.09 | 0.912 | 0.95 | 0.87, 1.03 | 0.109 |
| E | **0.89** | **0.80, 0.99** | **0.006** | **0.76** | **0.68, 0.84** | **<0.001** |
| *Dependent children (under 18) in household* | | | | | | |
| Yes | 0.98 | 0.90, 1.05 | 0.421 | 0.94 | 0.87, 1.01 | 0.028 |
| Number of adults in household | | | | | | |
| 1 | - | - | - | - | - | - |
| 2 | **1.08** | **1.01, 1.15** | **0.002** | **1.16** | **1.09, 1.24** | **<0.001** |
| 3 | **1.15** | **1.05, 1.26** | **<0.001** | **1.17** | **1.07, 1.28** | **<0.001** |
| 4 | **1.14** | **1.02, 1.28** | **0.004** | 1.08 | 0.96, 1.22 | 0.077 |
| 5 | 1.11 | 0.92, 1.34 | 0.152 | 1.03 | 0.85, 1.25 | 0.709 |
| 6 | 1.06 | 0.88, 1.28 | 0.432 | 1.00 | 0.82, 1.20 | 0.952 |

Bold indicates a significant result. *Alpha set to 0.01; 1. Bangladeshi, Chinese, Indian, Pakistani, and Asian – other background; 2. African, Caribbean, and Black – other background; 3. Mixed White & Asian, Mixed White & Black African, Mixed White & Black Caribbean, and Mixed – other background; 4. White British, White Irish, and White – other background.

Abbreviations: CI, confidence interval; OR, odds ratio.

Source: University of Sheffield Analysis using Kantar’s Worldpanel Take Home Purchase panel 2018 and 2021.

**Supporting Materials 5**

This sensitivity analysis was completed to assess the value chosen for determining occasional NoLo purchases (≥ 4 purchases per year). This was tested against ≥ 3 and ≥ 6 purchases of NoLo per year. Purchasing NoLo ≥ 3 times per year was significantly associated with increased weekly alcohol purchase at lower, increasing and higher risk purchasing levels; more adults living within a household (2, 3 and 4); and was less likely in households in social grades C2, D and E. These results are consistent with a purchasing threshold of ≥ 4 times per year. The analysis using a purchasing threshold of ≥ 6 times per year found comparable results, but this also found 5 adults living within a household were more likely to be NoLo purchasers than a single person household. Meanwhile social grade E was the only group less likely to purchase NoLo, however grades C2 and D were close to significance, suggesting lack of statistical power.

*Table S5: Sensitivity analysis using binary logistic regression exploring characteristics of households purchasing NoLo in 2021 to test different cut offs for households regularly purchasing NoLo*

|  | **NoLo purchase of ≥ 3 times per year** | | | **NoLo purchase of ≥ 6 times per year** | | |
| --- | --- | --- | --- | --- | --- | --- |
| **Predictor** | **OR** | **99% CI** | **p-value*** | **OR** | **99% CI** | **p-value*** |
| Constant | <0.01 | <0.01, 0.02 | <0.001 | <0.01 | <0.01, 0.01 | <0.001 |
| *Age of main shopper* | | | | | | |
| 18-28 | - | - | - | - | - | - |
| 28-34 | 1.07 | 0.27, 4.26 | 0.895 | 0.59 | 0.12, 2.82 | 0.384 |
| 34-44 | 0.98 | 0.26, 3.63 | 0.966 | 0.69 | 0.16, 2.93 | 0.507 |
| 45-54 | 0.97 | 0.26, 3.63 | 0.958 | 0.62 | 0.14, 2.67 | 0.399 |
| 55-64 | 0.87 | 0.23, 3.33 | 0.794 | 0.56 | 0.13, 2.48 | 0.314 |
| 65+ | 0.82 | 0.22, 3.11 | 0.702 | 0.47 | 0.11, 2.07 | 0.190 |
| *Ethnicity of main shopper* | | | | | | |
| Asian^1^ | - | - | - | - | - | - |
| Black^2^ | 3.82 | 1.00, 14.58 | 0.010 | 4.31 | 0.72, 25.66 | 0.035 |
| Mixed^3^ | 3.67 | 0.95, 14.13 | 0.013 | 1.07 | 0.12, 9.78 | 0.940 |
| White^4^ | 2.30 | 0.90, 5.91 | 0.023 | 2.39 | 0.75, 7.60 | 0.053 |
| Other | 4.95 | 0.73, 33.47 | 0.031 | 6.03 | 0.46, 80.05 | 0.073 |
| Unknown | 2.24 | 0.74, 6.80 | 0.061 | 2.07 | 0.49, 8.82 | 0.197 |
| *Region of household* | | | | | | |
| England | - | - | - | - | - | - |
| Scotland | 1.17 | 0.86, 1.60 | 0.187 | 1.19 | 0.79, 1.78 | 0.278 |
| Wales | 1.13 | 0.71, 1.79 | 0.496 | 0.79 | 0.42, 1.50 | 0.343 |
| *Social grade* | | | | | | |
| AB | - | - | - | - | - | - |
| C1 | 0.89 | 0.71, 1.12 | 0.187 | 0.91 | 0.67, 1.24 | 0.425 |
| C2 | **0.71** | **0.53, 0.94** | **0.002** | 0.71 | 0.49, 1.04 | 0.022 |
| D | **0.58** | **0.41, 0.84** | **<0.001** | 0.63 | 0.39, 1.02 | 0.013 |
| E | **0.44** | **0.27, 0.71** | **<0.001** | **0.46** | **0.24, 0.86** | **0.001** |
| *Weekly alcohol purchase per adult* | | | | | | |
| Non-purchaser | - | - | - | - | - | - |
| Low risk (≤112g per week) | **7.69** | **3.03, 19.50** | **<0.001** | **9.73** | **2.91, 32.46** | **<0.001** |
| Increasing risk (>112 to ≤280g per week) | **11.58** | **4.47, 30.00** | **<0.001** | **18.35** | **5.37, 62.70** | **<0.001** |
| Higher risk (>280g per week) | **13.64** | **5.01, 37.15** | **<0.001** | **17.62** | **4.86, 63.87** | **<0.001** |
| *Dependent children (under 18) in household* | | | | | | |
| Yes | 1.01 | 0.76, 1.34 | 0.949 | 0.91 | 0.63, 1.32 | 0.510 |
| Number of adults in household | | | | | | |
| 1 | - | - | - | - | - | - |
| 2 | **1.77** | **1.34, 2.34** | **<0.001** | **2.10** | **1.43, 3.09** | **<0.001** |
| 3 | **1.64** | **1.17, 2.31** | **<0.001** | **1.83** | **1.14, 2.94** | **0.001** |
| 4 | **1.84** | **1.13, 3.00** | **0.001** | **2.62** | **1.35, 5.09** | **<0.001** |
| 5 | 1.86 | 0.93, 3.72 | 0.021 | **3.15** | **1.34, 7.41** | **0.001** |
| 6 | 1.33 | 0.60, 2.96 | 0.354 | 1.96 | 0.71, 5.45 | 0.089 |

Bold indicates a significant result. *Alpha set to 0.01; 1. Bangladeshi, Chinese, Indian, Pakistani, and Asian – other background; 2. African, Caribbean, and Black – other background; 3. Mixed White & Asian, Mixed White & Black African, Mixed White & Black Caribbean, and Mixed – other background; 4. White British, White Irish and White – other background.

Abbreviations: CI, confidence interval; OR, odds ratio.

Source: University of Sheffield Analysis using Kantar’s Worldpanel Take Home Purchase panel 2018 and 2021

**Supporting Materials 6**

Process for creating ‘number of adults in household’ variable

We wanted to create *number of adults in household* as a variable to use as a control variable and then to calculate how much alcohol was purchased by each adult in the household. Kantar’s Worldpanel (KWP) Take Home data contains a variable called household size (1-5+) and a variable called number of children (0-3+). We used household size minus the number of children to determine the number of adults. We recognised that larger households would not be reflected, so we imputed the following scenarios to address this:

- In situations where there were zero children and five people in the household, we evenly split these households in to having 5 or 6 adults.
- In situations where there was one child and five people in the household, we evenly split these households in to having 4, 5 or 6 adults.
- In situations where there were two children and five people in the household, we evenly split these households in to having 3, 4, 5 or 6 adults.
- In situations where there were three children and five people in the household, we evenly split these households in to having 1, 2, 3, 4, 5 or 6 adults.
